# Structural Determination of the Australian Bat Lyssavirus Nucleoprotein and Phosphoprotein Complex

**DOI:** 10.3390/v16010033

**Published:** 2023-12-23

**Authors:** Camilla M. Donnelly, Murray Stewart, Justin A. Roby, Vinod Sundaramoorthy, Jade K. Forwood

**Affiliations:** 1 School of Dentistry and Medical Sciences, Charles Sturt University, Wagga Wagga, NSW 2678, Australia; 2 Gulbali Institute, Charles Sturt University, Wagga Wagga, NSW 2678, Australia; 3 Diagnostics, Surveillance and Response, Australian Centre for Disease Preparedness, CSIRO, Geelong, VIC 3219, Australia; 4 MRC Laboratory of Molecular Biology, Francis Crick Ave., Cambridge Biomedical Campus, Cambridge CB2 0QH, UK; 5 School of Medicine, Deakin University, Geelong, VIC 3216, Australia

**Keywords:** rhabdovirus, Australian bat lyssavirus, rabies virus, phosphoprotein, nucleoprotein, nucleocapsid, X-ray crystallography, small-angle X-ray scattering

## Abstract

Australian bat lyssavirus (ABLV) shows similar clinical symptoms as rabies, but there are currently no protein structures available for ABLV proteins. In lyssaviruses, the interaction between nucleoprotein (N) and phosphoprotein (N) in the absence of RNA generates a complex (N^0^P) that is crucial for viral assembly, and understanding the interface between these two proteins has the potential to provide insight into a key feature: the viral lifecycle. In this study, we used recombinant chimeric protein expression and X-ray crystallography to determine the structure of ABLV nucleoprotein bound to residues 1–40 of its phosphoprotein chaperone. Comparison of our results with the recently generated structure of RABV CVS-11 N^0^P demonstrated a highly conserved interface in this complex. Because the N^0^P interface is conserved in the lyssaviruses of phylogroup I, it is an attractive therapeutic target for multiple rabies-causing viral species.

## 1 Introduction

Rabies disease is caused by viruses belonging to the genus *Lyssavirus*, which contains 17 member species that are bullet-shaped, single-stranded, negative-sense RNA viruses (order *Mononegavirales*, family *Rhabdoviridae)*, with the prototypical species being *Lyssavirus rabies* (RABV). Rabies is a zoonotic disease responsible for approximately 60,000 human deaths every year (World Health Organization, 2021) and has one of the highest case-fatality rates of infectious diseases at almost 100%. There is no effective treatment available after the onset of neurological symptoms. Despite the entire genus being responsible for causing rabies disease, much of the existing research focus has been on *Rabies lyssavirus*, and little is known about the diversity across the genus.

Although the continent of Australia is free from RABV, a rabies-causing *Australian bat lyssavirus* (ABLV) was identified in an encephalitic bat in 1996 [1]. The zoonotic capability of ABLV was reported soon after, with the hospitalisation and death of a wildlife carer who sustained scratches from flying foxes [2]. ABLV is clinically indistinguishable from RABV infection. Human cases are rare, with a total of three human fatalities from ABLV. ABLV can also infect domestic animals, with two horses diagnosed in 2013 [3], and experimentally infected cats and dogs displayed mild behavioural changes and sero-conversion [4]. Bats suffering illness from ABLV infection are more likely to come into contact with humans [5], and wildlife carers are at increased risk of exposure. Clusters of ABLV in Pteropodid bats are known to occur [6], and several sick bats have been identified with ABLV in recent months [7,8].

Two distinct strains of ABLV have since been described in Australian bats, with the initial strain being of the Pteropodid bat strain (flying foxes) and the second from insectivorous bats, first detected in the Yellow-bellied sheathtail bat (*Saccolaimus flaviventris*). Screening of Australian bat populations has shown ABLV in five out of six bat families present, and widespread prevalence [9]. As such, all contact with bats should be considered a potential transmission event, and exposed individuals should receive post-exposure prophylaxis in the form of a rabies vaccine and hIG as these species are in the same lyssavirus phylogroup, and cross-reactivity is present [10,11].

The lyssavirus genome is approximately 12 kb, with ABLV having 11,918 nucleotides that encode five genes for the corresponding proteins [12]. In *Mononegavirales*, the nucleoprotein (N protein, known as NP in *Filoviridae*) is an RNA-binding protein that encapsidates the viral RNA genome [13], with each protomer binding to nine nucleotides [14] and forming a helical homo-oligomer nucleocapsid to encapsidate the entire single RNA strand [14]. Encapsidation prevents viral RNA recognition by RNA-detecting pattern recognition receptors of the cell’s innate immune system [15]. The RABV and ABLV nucleoproteins contain 450 residues, with RABV nucleoprotein being phosphorylated by the host at Ser^389^ [16], which is necessary for transcription and replication [17,18]. For RABV, strain-specific differences in nucleoprotein sequences have been linked to differences in pathogenicity in the brain, allowing the virus to evade the innate immune response [19,20]. Furthermore, the N-RNA template binds to the RNA-dependent RNA polymerase (L protein) and its non-catalytic cofactor phosphoprotein (P protein) to form ribonucleoprotein (RNP).

Lyssavirus infection is typified by large cytoplasmic inclusions in host cells. These cytoplasmic inclusions are known as Negri bodies, contain concentrated RNP, and serve as viral factories in infection [21]. Negri bodies arise through a concentration of low-affinity interactions between proteins and nucleic acids, driving liquid–liquid phase separation. It is thought that this allows viral proteins to be protected from intracellular pathways of detection (pattern recognition receptors). The cell culture expression of the RABV N and P proteins is sufficient to produce Negri-like bodies in the cytoplasm. Within the Negri bodies, the phosphoprotein also binds as a chaperone to newly synthesised nucleoprotein. During viral replication, a constant supply of soluble nucleoprotein is required to encapsidate newly synthesised RNA (reviewed in [22]). The phosphoprotein acts as a chaperone to keep the nucleoprotein soluble, prevent self-association, and to maintain it in a conformation ready for its addition to the newly synthesised viral RNA [23,24]. The co-expression of N and P proteins generates a complex that confers specificity for viral RNA, whereas the expression of the nucleoprotein in the absence of phosphoprotein binds mRNA non-specifically [25]. This interaction is mediated by phosphoprotein residues 4–40 in the N-terminal region (NTR) [24].

The secondary structure of the N-terminal region is predicted to be helical [24], and this is supported by nuclear magnetic resonance (NMR) analysis of the VSV phosphoprotein that shows two transient helices in the NTR [26]. Structural characterisation of the N^0^P interaction from other *Mononegavirales*, including the Rhabdovirus vesicular stomatitis virus (VSV), shows a peptide containing residues 1–60 of the phosphoprotein as the chaperone module that changes dynamically from a disordered region to a highly ordered helix on binding to the N protein [26]. The N-terminus of the RABV phosphoprotein was also analysed using an array of disorder prediction software and was predicted to be structured from residues 1 to 29 [27], which would allow the P protein to bind the N protein to prevent N-N interactions while keeping the positively charged RNA-binding site open. The stoichiometry of the N^:^P interaction in the N^0^P complex formed in the absence of RNA was established using nuclear magnetic resonance (NMR) and small-angle x-ray scattering (SAXS) and was found to be one dimer of P bound to two N^0^ molecules, giving a mixture of 1N^0^:2P or 2N^0^:2P [28] (Yabukarski et al., 2016). This ratio suggests that the phosphoprotein is operating as a dimer, enabling it to adopt a conformation that simultaneously solubilises two N proteins.

To date, no protein structures from ABLV proteins have been described. The importance of the N^0^P interface in the lifecycle of the lyssavirus and its prerequisite to viral assembly makes it an attractive target for therapeutic intervention. Understanding the N^0^P interface has the potential to provide a target for antivirals, as this step is critical in producing viral progeny. Furthermore, elucidation could provide an important basis for the design of therapeutics for lyssaviruses. We used the previously successful chimeric approach to express and crystallize ABLV nucleoprotein and the chaperone module of phosphoprotein (1–53). Here, we present the crystal structure of the ABLV nucleoprotein-phosphoprotein (1–53) interface. We provide a structural comparison with RABV CVS-11 N^0^P [29] and describe a conserved interface. The highly conserved interface of N^0^P of these phylogroup I lyssaviruses (and potentially for broader rhabdoviruses) could lend itself as a broad therapeutic target.

## 2 Materials and Methods

### 2.1 Plasmids

The expression construct was designed as a chimera to encode residues 1–53 of the phosphoprotein, a TEV protease site linker, then the full-length (residues 1-450) nucleoprotein from GenBank AF006497.1 Australian Bat Virus; lyssavirus (Ballina isolate). Accession numbers for the N and P proteins are AAD01267.1 and AAD0168.1, respectively. The sequence was optimised for *E. coli* expression and cloned into the pET30(a) vector at the BamHI/BamHI site by GenScript. The plasmid also encoded an N-terminal 6His tag and TEV protease cleavage site, with the overall protein having the architecture 6His-TEV-P(1–53)-TEV-N(1–450). In the chimeric protein, residues 1–50 are His tag, 51–57 TEV site, 58–110 are P(1–53), 111–117 are TEV site, and 118–567 are N(1–450), but, to avoid confusion, amino acids will be referred to by the position in the native proteins and not the chimera construct.

### 2.2 Protein Expression and Purification

The ABLV N^0^P plasmid was transformed into chemically competent BL21 (DE3) pLysS cells using heat shock [30]. Transformed colonies were selected with 50 μg/mL of kanamycin, and the recombinant protein expression was induced by 500 mM Isopropyl-β-D-thiogalactoside (IPTG) at 16 °C for 15 h [31]. Cells were harvested by centrifugation at 5400× *g* and the cell pellets were resuspended in His buffer A (50 mM phosphate buffer, 300 mM sodium chloride, 20 mM imidazole, pH 8.0) and frozen at −20 °C for future use. Cells underwent two freeze–thaw cycles and were lysed with 1 mL of lysozyme (20 mg/mL) with the addition of DNase (5 μg/mL) and incubated for 45 min at room temperature. The whole cell extract was passed through a 22-gauge needle to shear any remaining DNA or cell clumps before centrifugation at 11,800× *g* at 16 °C for 30 min. The soluble extract was then filtered with a 0.45 μm low protein affinity filter before purification using immobilised metal affinity chromatography (IMAC) with a 5mL HisTrap HP (Cytiva), pre-equilibrated with His A buffer. The target protein was eluted using a gradient of His B buffer (500 mM imidazole, 300 mM sodium chloride, 50 mM phosphate buffer). A sub-sequent step of size exclusion chromatography was performed on the Superdex 200 pg 26/600 column (GE Healthcare) using Tris-buffered saline (50 mM Tris-HCl, 125 mM sodium chloride, pH 8.0). A small sample of purified protein was treated with TEV protease to confirm the expression of the ABLV N^0^P protein. The protein fractions were analysed with SDS-PAGE, pooled, and concentrated with a 10 kDa centrifugal filter to 30 mg/mL.

### 2.3 Protein Crystallisation, Data Collection, and Processing

The purified ABLV N^0^P protein was used in crystallisation trials using the hanging-drop vapour diffusion method in 48-well plates using 1.5 μL of protein combined with 1.5 μL crystallant solution and equilibrated over a reservoir containing 300 μL of crystallant solution. The ABLV N^0^P chimera formed clusters of needle-like crystals that appeared in screens after four weeks at 30 mg/mL at 23 °C. The high-resolution atomic structure of the ABLV N^0^P dataset was collected from the nominate crystal grown in Morpheus 1.45 from Molecular Dimensions (0.12 M alcohols mix (0.1 M 30% *v*/*v* 0.2 M 1,6-Hexanediol; 0.2 M 1-Butanol, 0.2 M 1,2-Propanediol; 0.2 M 2-Propanol; 0.2 M 1,4-Butanediol; 0.2 M 1,3-Pro-panediol), 1.0M Tris (base) pH 8.5; BICINE, 60% precipitant mix (40% *v*/*v*/PEG 500 MME; 20% *w/v* PEG 20000).

Crystals were cryoprotected and flash-cooled in liquid nitrogen before data X-ray diffraction data were collected at the Australian Synchrotron on the MX2 beamline using a Dectris Eiger 16M detector. Data reduction and integration were performed using XDS and scaled using Aimless [32] before molecular replacement in PhaserMR [33] using the 2.0 Å resolution in-house structure of the RABV N^0^P. Several rounds of model building and refinement were performed in COOT [34] and Phenix [35] to complete the molecular model. Protein–protein interactions were analysed by PDBSUM (See supplementary Table S1) [36] and compared to PDB 8B8V using CCP41 GEMSANT. Data collection and refinement statistics are given in Table 1.

## 3 Results

### 3.1 The ABLV Phosphoprotein Chaperone Module Binds to the RNA-Free Nucleoprotein

**Figure 1:**
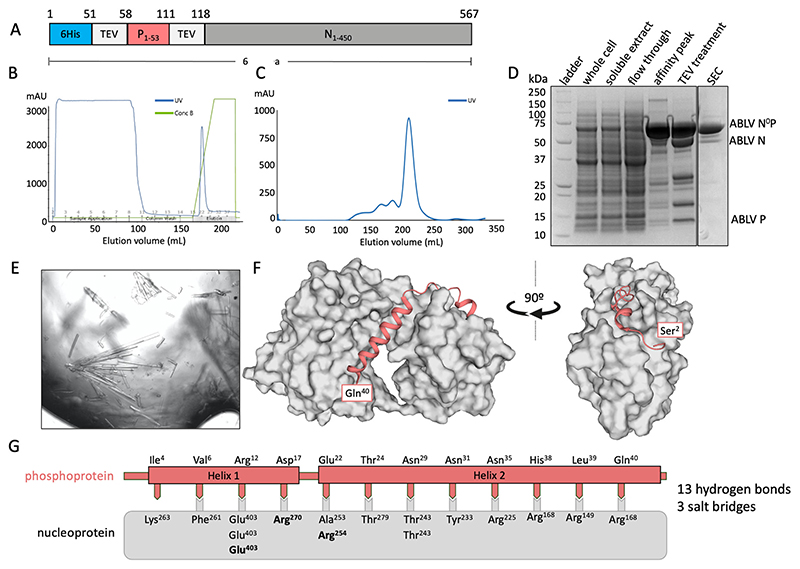
X-ray crystal structure of ABLV nucleoprotein (grey) bound to its phosphoprotein chaperone in an RNA-free state. (**A**) Schematic of expressed protein containing an N-terminal 6His tag followed by a TEV protease site for cleavage, the phosphoprotein fragment 1–53, a second TEV protease site, and the full-length nucleoprotein. (**B**) Chromatograph of affinity purification of ABLV N^0^P using IMAC. A single elution peak was obtained. (**C**) Chromatograph of subsequent size-exclusion chromatography (SEC) on FPLC. A large symmetrical peak was eluted from 200 to 230 mL. (**D**) SDS-PAGE of ABLV N^0^P purification showing purified target protein at approximately 64 kDa in affinity peak and SEC. Treatment with TEV protease resulted in cleavage and confirmation that the purified protein was ABLV N^0^P. (**E**) The ABLV N^0^P structure was obtained using crystals generated in crystallisation condition Morpheus 1–45 from Molecular Dimensions. (**F**) The resolved ABLV N^0^P structure. The phosphoprotein chaperone adopted a helix–loop–helix conformation and bound to hydrophobic grooves on the nucleoprotein (grey surface representation). The nucleoprotein adopted a typical jaw-like globular structure. Figure generated in Pymol. (**F**) Schematic of the interactions between the ABLV nucleoprotein (grey box) and phosphoprotein (salmon). Interfacing residues are indicated by text, with salt bridges in bold.

A chimeric fusion protein containing residues 1–60 of the phosphoprotein and the full-length (residues 1–450) nucleoprotein was used to determine the binding interface of ABLB N^0^P (Figure 1A). The chimeric protein was purified using IMAC (Figure 1B) and subsequently eluted from a Superdex 200 size-exclusion column as a large, single peak and showed no RNA contamination (A260/280 = 0.7) (Figure 1C). Its composition was confirmed using SDS-PAGE with TEV protease cleavage (Figure 1D). Only trace amounts of protein degradation were detected.

The chimeric protein was used to generate crystals that had *P2* symmetry and which diffracted to a resolution of 2.19 Å and had unit cell parameters of a = 82.37 Å, b = 35.50 Å, and c = 89.09 Å, with *α* = 90°, β = 92.62°, and γ = 90°. There was a single molecule of the N^0^P chimera in the asymmetric unit. The final model had excellent stereochemistry and R_work_ and R_free_ of 16% and 19%, respectively. The model was deposited into the Protein Data Bank with the code 8FWL. Full data collection and refinement statistics are presented in Table 1.

Residues 27–447 of the nucleoprotein could be traced, except for a chain break between residues 352 and 399 that remained unresolved due to its being in a flexible loop. The structure of nucleoprotein contained the N-terminal and C-terminal globular domains that contained multiple alpha helices together with a central hinge region that resulted in it having the jaw-like structure typical of *Mononegavirales* nucleoproteins [14,23,29]. The phosphoprotein chaperone module had good electron density and could be traced from residues 2 to 40. Its N-terminus appeared to be essentially unstructured and was followed by two helical domains (Helix 1 Pro^8^-Arg^12^ and Helix 2 Met^20^-Gln^40^) that were separated by an unstructured loop. There was no electron density that corresponded to phosphoprotein residues 41–53 or the TEV site linker. The crystal structure also contained 172 water molecules and one PEG molecule derived from the crystallisation solution (Figure 1F).

The structure indicated that the interaction between the phosphoprotein and the nucleoprotein is mediated by 13 H-bonds and three salt bridges, with Helix 2 of the phosphoprotein being amphipathic and forming a large number of hydrophobic interactions within the binding groove of the nucleoprotein [36].

### 3.2 The N^0^P Interface Is Highly Conserved between ABLV and RABV CVS-11

**Figure 2:**
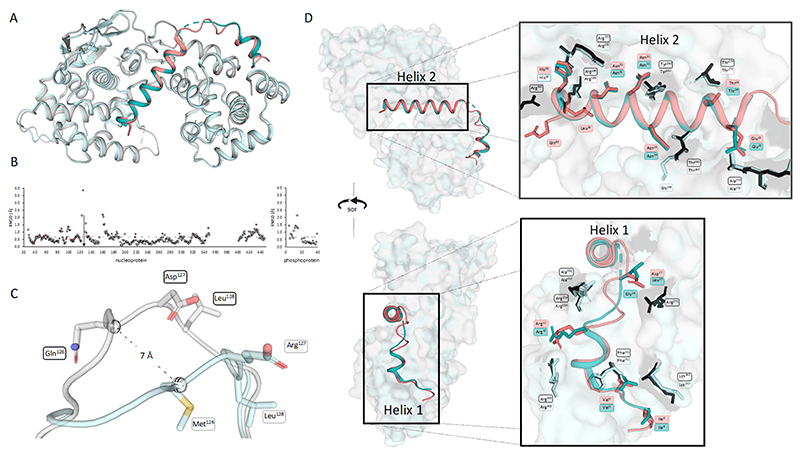
Comparison of ABLV N^0^P from this study with RABV N^0^P showed a conserved structure. (**A**) Aligned structures of ABLV N^0^P (nucleoprotein is grey and phosphoprotein is salmon) and RABV CVS-11 N^0^P (PDB 8B8V) (nucleoprotein light cyan and phosphoprotein teal) show a conserved structure. (**B**) ABLV N^0^P and RABV N^0^P were compared using CCP4i2 GESAMT [37]. Graphs showing the difference in position for each amino acid is measured in Root Mean Square of Deviation (RMSD) Å and plotted against the amino acid number for N (left) and P (right). Differences in amino acid sequence are shown in salmon-coloured markers. The average RMSD for the N^0^P complex is shown as a dotted line. (**C**) Pymol alignment of ABLV and RABV nucleoprotein loop region (residues 122–130) showing the 7 Å difference in position of ABLV ^126^QDL^128^ and RABV ^126^MEL^128^. (**D**) Image of aligned N^0^P structures showing the conserved interfacing residues as sticks (ABLV phosphoprotein in salmon, nucleoprotein in black, RABV nucleoprotein cyan, phosphoprotein in teal). Figure generated using Pymol.

The crystal structure of ABLV N^0^P was compared to the recently published crystal structure of RABV CVS-11 N^0^P (PDB 8B8V [29]). The fold of the nucleoprotein and positioning of the phosphoprotein chaperone modules were conserved in the two N^0^P structures (Figure 2A). The structures were analysed using CCP4i2 GESAMT and showed an overall RMS difference of 0.6Å, demonstrating the high degree of similarity between the two structures. The distance (Å) of each equivalent residue was plotted for both the N and P proteins (Figure 2B). The flexible loop region (residues 113–130) showed the greatest variation between the ABLV and RABV nucleoproteins. This flexible loop does not have equivalent residues, with the ABLV motif being ^126^QDL^128^ and RABV CVS-11 ^126^MEL^128^; hence, CCP4i2 GESAMT did not generate data points on the graphs (Figure 2C). Pisa server analysis shows that the hydrophobic RABV Met^126^ is 80% buried and forms a hydrogen bond with the zeta nitrogen of nucleoprotein Lys^54^, whereas ABLV Gln^126^ is hydrophilic and solvent-accessible and does not interact with internal nucleoprotein residues, and its alpha carbon was positioned 7 Å away from that of RABV Met^126^.

The ABLV phosphoprotein chaperone Helix 2 occluded the RNA-binding site and interacted with RNA-binding residues Arg^168^, Arg^149^, and Arg^225^. It is inferred that the phosphoprotein chaperone module prevents nucleoprotein oligomerisation in ABLV based on its relative position and occlusion of N:N interaction surfaces in the RABV oligomeric N protein structure [14,29]. Nucleoprotein residues Arg^168^ and Arg^149^ have also been implicated in the formation of biocondensates and phase separation in the presence of phosphoprotein [38]. Phosphoprotein Helix 1 binds at the same site as the adjacent nucleoprotein protomers of RABV. Phosphoprotein Arg^12^ forms two hydrogen bonds and a salt bridge with nucleoprotein Glu^403^, which would otherwise bind N-1 Arg^357^ and Arg^361^. Additionally, the phosphoprotein Helix 2 provides major clashes at the binding site of N+1 N-terminus (Ile^6^-Gln^25^), but overall, the binding interface of N^0^P was conserved between the two lyssavirus species.

## 4 Discussion

We have used recombinant protein expression and X-ray crystallography to obtain the first protein structures for ABLV. Using a chimera of phosphoprotein residues 1–53 and full-length nucleoprotein, we purified and crystallised the protein and solved the structure to 2.19 Å resolution. The structure showed that the chaperone module of phosphoprotein is comprised of two amphipathic alpha helices that bind to a single nucleoprotein protomer along a hydrophobic groove consistent with a range of studies that implicate the lyssavirus phosphoprotein N-terminus in interactions with nucleoprotein. [23,24].

The chimeric approach of fusing the N-terminus of the P protein to the full-length N protein was employed to improve the stability of the N^0^P protein complex. The validity of this approach is supported by its use in similar studies. For investigations into EBOV N^0^P, the nucleoprotein copurified with VP35 N-terminal peptide (P equivalent) and was pulled down in a 1:1 ratio. However, the complex shifted to a higher-molecular-weight species over time [39]. Therefore, an N-VP35 chimera was successfully employed for structural determination. This approach was also taken for MeV N^0^P, and it was found that it prevented the nucleoprotein encapsidation of non-specific *E. coli* RNA during the expression process [40]. Renner also used this strategy for human metapneumovirus N^0^P [41]. Therefore, this established approach was adopted for ABLV N^0^P crystallography.

The interface between the phosphoprotein and nucleoprotein in the ABLV N^0^P chimera in the present study is supported by the recent publication of the RABV (CVS-11 strain) N^0^P protein [29]. These authors chose to express phosphoprotein 1–68 and nucleoprotein 24–450 independently, and the complex was confirmed by SEC-MALLS prior to crystallography [29]. The truncation of the nucleoprotein prevented polymerisation mediated through the N-terminal arm. Despite the two different approaches to generating the N^0^Ps, the ABLV and RABV crystal structures have a high similarity (RMS 0.69), giving confidence that the ABLV structure was unaffected by the engineered linker regions. Both the ABLV and RABV structures lacked electron density for the flexible nucleoprotein C-terminal arm (residues 352–399 and 350–400 for ABLV and RABV, respectively). It is, therefore, likely that this motif is also critical in the docking of N subunits for polymerisation and the formation of the nucleocapsid [14].

**Figure 3:**
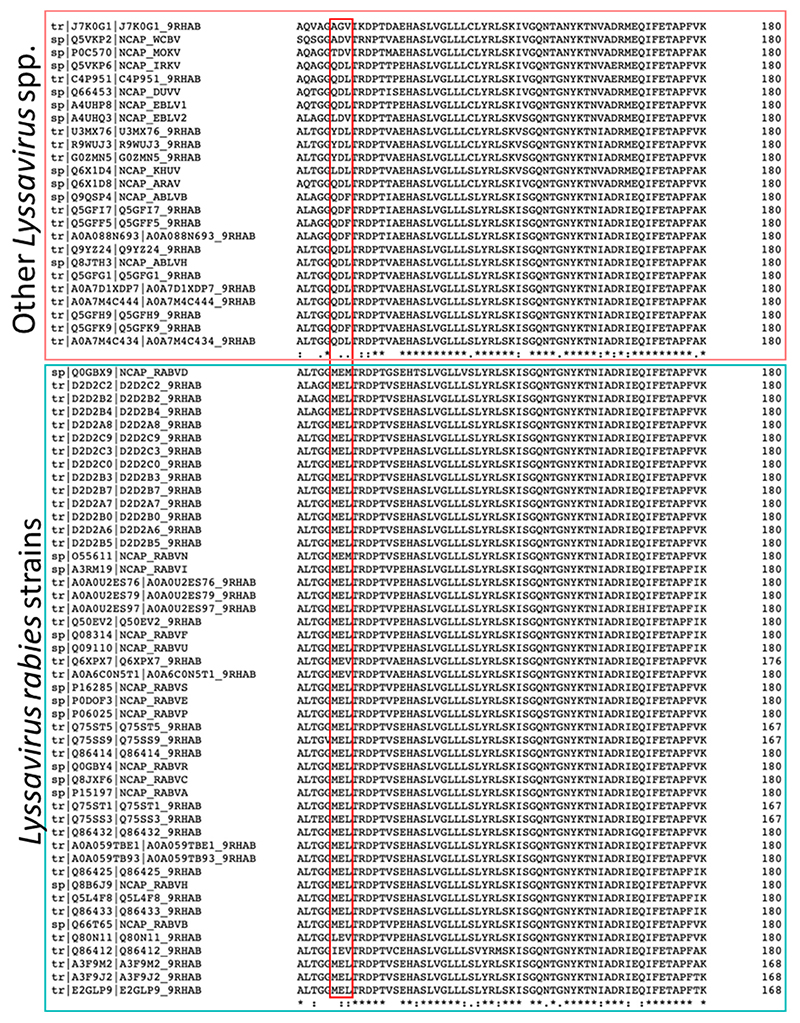
The alignment of positions 121–180 of the nucleoprotein sequences in Lyssaviruses shows a high conservation of the ^126^MEL^128^ motif between RABV strains (indicated by the red box in the bottom panel). However, non-RABV lyssaviruses have variations in the ^126^QDL^128^ motif (indicated by the red box in the top panel). The alignment was conducted using the Clustal Omega Multiple sequence alignment tool [45].

The overall molecular architecture of the nucleoprotein and phosphoproteins and the interface between them in the N^0^P complex were conserved, consistent with the similarity of their amino acid sequences (92% for nucleoproteins and 85.9% for phosphoproteins), with only one amino acid substitution existing in the observed residues of the phosphoprotein (ABLV Met^15^, RABV Leu^15^). However, there were significant differences between ABLV and RABV in nucleoprotein residues 126–128, which has three substitutions. A sequence alignment of lyssavirus nucleoprotein sequences showed that the ^126^MEL^128^ motif is highly conserved amongst RABV strains, in contrast to non-RABV lyssaviruses that show high similarity to the ABLV ^126^QDL^128^ motif (Figure 3). The substitutions may play a role in the nucleocapsid assembly with the matrix protein (M), and there may be subtle differences between species. This hypothesis is supported by research on the VSV nucleoprotein which shows that the equivalent loop on the VSV nucleoprotein binds to the VSV matrix protein [42,43]. Current knowledge about the structure of lyssavirus matrix protein is limited to that from *Lyssavirus lagos* (LBV) [44].

Research into lyssaviruses has been mostly restricted to RABV, and often to laboratory-adapted strains. In RABV, the phosphoprotein is expressed both as a full-length (P1) protein and also as four progressively N-terminally truncated isoforms (P2–P5) though ribosomal leaky scanning and alternative start codons. All these phosphoproteins exist as homodimers and contain a mixture of structured and intrinsically disordered regions (IDRs) [46,47]. ABLV, in contrast, is translated into three isoforms with a full-length P1 together with P2 (residues 20–97) and a P5 equivalent (residues 83–297) because the Met53Ile and Met69Asn substitutions in ABLV are not consistent with alternate initiation sites. The RABV phosphoprotein has been shown to be phosphorylated at a number of sites, including Ser63 and Ser64, by RABV protein kinase and Ser162, Ser210, and Ser271 by protein kinase C isomers [48]. Because ABLV also has serines at positions 63, 210, and 271, it may also be phosphorylated in a similar manner. Although, clearly, further research is needed to understand non-RABV lyssaviruses like ABLV, there may be differences because when compared in terms of pathogenicity and replication kinetics, ABLV grows more slowly than RABV, and the incubation period can be as long as 27 months [49,50].

Lyssavirus phosphoproteins have several functions in addition to acting as a nucleoprotein chaperone. The N-terminal region (NTR—residues 1–19) has been identified as the interaction site for the C-terminus of the RNA-dependent RNA polymerase (RdRp, the L protein), where phosphoprotein acts as a RdRp cofactor [51,52]. Curiously, this interface is only present in the P1 isoform because it is absent in the truncated P2-P5 isoforms, suggesting that only full-length P functions as a polymerase cofactor. In contrast to this study, the L-P binding interface determined by cryoEM [53] shows that a fragment of phosphoprotein (residues 1–91) was sufficient to bind to the L protein. A total of 27 phosphoprotein residues are described as binding to L (between residues 51 and 87). A possible salt bridge is formed via P51, which extends the knowledge of the interface involved in polymerase activity. Consequently, there is a dual function for the phosphoprotein NTR as this is also the region responsible for chaperone functions, as confirmed structurally for ABLV and RABV [29].

Lyssavirus P proteins also perform accessory roles, with the C-terminal domain (CTD) being extensively described as an antagonist to type 1 interferon (IFN) response. Several studies have demonstrated that phosphoprotein interferes with IFN induction by blocking IRF-3 phosphorylation through the kinases TBK-1 and IKK∈ [54–57]. Furthermore, RABV phosphoprotein CTD inhibits downstream IFN signalling by interacting with phosphorylated STAT-1, STAT-2, and STAT-3. This interaction causes the accumulation of STATS in the cytoplasm, thereby blocking IFN signalling and failing to induce a robust host antiviral response [58–61]. Binding sites for host proteins, including PML, microtubules, STATs, and nuclear import/export machinery, have all been attributed to the phosphoprotein CTD [62–65]. The role of these regions has been the topic of much study, with strong evidence that these isoforms also exist as innate immune system modulators [65]. It is unknown whether functions of the phosphoprotein NTR and CTD are simultaneous, sequential, mutually exclusive, or undertaken by discrete phosphoprotein molecules.

## 5 Conclusions

This study has determined the structure of ABLV nucleoprotein and the interface with its phosphoprotein chaperone in the absence of RNA. The findings here suggest that there is a highly conserved interface between ABLV and RABV CVS-11 N0P structures, which could be potentially targeted for therapeutic purposes against these, and possibly multiple other rabies-causing species, and contribute to the development of effective treatments for both ABLV and a broad range of lyssaviruses.

## Supplementary Material

Supplementary Material - Table S1

## Figures and Tables

**Table 1:** Data collection and refinement statistics.

Data Collection and Processing	ABLV N^0^P
Wavelength (Å)	0.95365
Resolution range (Å)	29.67–2.19 (2.26–2.19)
Space group	P 1 2 1
Unit cell (Å, °)	82.37 35.50 89.09 90 92.63 90
Total reflections	102,224 (8160)
Unique reflections	26,863 (2185)
Multiplicity	3.8 (3.7)
Completeness (%)	99.5 (94.8)
Mean I/sigma(I)	10.4 (3.3)
Wilson B-factor Å^2^	29.119
R-merge	0.076 (0.464)
R-pim	0.044 (0.268)
CC1/2	0.997 (0.866)
Refinement	
Number of reflections	26,858 (2300)
Number of R-free reflections	1371 (143)
R-work %	0.1594 (0.2028)
R-free %	0.1925 (0.2277)
RMS(bonds)	0.004
RMS(angles)	0.546
Ramachandran plot	
favoured (%)	98.53
allowed (%)	1.47
outliers (%)	0
Average B-factor Å^2^	45.56
Clash score	1.97
MolProbity score	0.70 (100%)
PDB accession code	8FWL

Statistics for the highest-resolution shell are shown in parentheses.

## Data Availability

Coordinate and structure factors have been deposited to the Protein Data Bank under the accession code 8FWL.

## References

[1] Fraser GC, Hooper PT, Lunt RA, Gould AR, Gleeson LJ, Hyatt AD, Russell GM, Kattenbelt JA (1996). Encephalitis caused by a Lyssavirus in fruit bats in Australia. Emerg Infect Dis.

[2] Murray K, Morgan J, Allworth A (1996). A human case of encephalitis due to a lyssavirus recently identified in fruit bats. Commun Dis Intell.

[3] Shinwari MW, Annand EJ, Driver L, Warrilow D, Harrower B, Allcock RJ, Pukallus D, Harper J, Bingham J, Kung N (2014). Australian bat lyssavirus infection in two horses. Vet Microbiol.

[4] McColl KA, Chamberlain T, Lunt RA, Newberry KM, Westbury HA (2007). Susceptibility of domestic dogs and cats to Australian bat lyssavirus (ABLV). Vet Microbiol.

[5] O’Connor T, Finlaison D, Kirkland P (2022). What can we learn from over a decade of testing bats in New South Wales to exclude infection with Australian bat lyssaviruses?. Aust Vet J.

[6] Barrett J, Höger A, Agnihotri K, Oakey J, Skerratt LF, Field HE, Meers J, Smith C (2020). An unprecedented cluster of Australian bat lyssavirus in Pteropus conspicillatus indicates pre-flight flying fox pups are at risk of mass infection. Zoonoses Public Health.

[7] Wuth R (2023). AAP General News Wire.

[8] Queensland Government, Queensland Health (2023). Public Health Alert: Confirmed ABLV in Flying Fox Found in Urangan.

[9] Field HE (2018). Evidence of Australian bat lyssavirus infection in diverse Australian bat taxa. Zoonoses Public Health.

[10] Weir DL, Coggins SA, Vu BK, Coertse J, Yan L, Smith IL, Laing ED, Markotter W, Broder CC, Schaefer BC (2021). Isolation and Characterization of Cross-Reactive Human Monoclonal Antibodies That Potently Neutralize Australian Bat Lyssavirus Variants and Other Phylogroup 1 Lyssaviruses. Viruses.

[11] Merritt T, Taylor K, Cox-Witton K, Field H, Wingett K, Mendez D, Power M, Durrheim D (2018). Australian bat lyssavirus. Aust J Gen Pract.

[12] Warrilow D, Smith IL, Harrower B, Smith GA (2002). Sequence Analysis of an Isolate from a Fatal Human Infection of Australian Bat Lyssavirus. Virology.

[13] Viral Zone Negative Stranded RNA Virus Replication.

[14] Albertini AA, Wernimont AK, Muziol T, Ravelli RB, Clapier CR, Schoehn G, Weissenhorn W, Ruigrok RW (2006). Crystal structure of the rabies virus nucleoprotein-RNA complex. Science.

[15] Šantak M, Matić Z (2022). The Role of Nucleoprotein in Immunity to Human Negative-Stranded RNA Viruses&Mdash; Not Just Another Brick in the Viral Nucleocapsid. Viruses.

[16] Dietzschold B, Lafon M, Wang H, Otvos L, Celis E, Wunner WH, Koprowski H (1987). Localization and immunological characterization of antigenic domains of the rabies virus internal N and NS proteins. Virus Res.

[17] Yang J, Koprowski H, Dietzschold B, Fu ZF (1999). Phosphorylation of Rabies Virus Nucleoprotein Regulates Viral RNA Transcription and Replication by Modulating Leader RNA Encapsidation. J Virol.

[18] Wu X, Gong X, Foley HD, Schnell MJ, Fu ZF (2002). Both Viral Transcription and Replication Are Reduced when the Rabies Virus Nucleoprotein Is Not Phosphorylated. J Virol.

[19] Masatani T, Ito N, Ito Y, Nakagawa K, Abe M, Yamaoka S, Okadera K, Sugiyama M (2013). Importance of rabies virus nucleoprotein in viral evasion of interferon response in the brain. Microbiol Immunol.

[20] Masatani T, Ito N, Shimizu K, Ito Y, Nakagawa K, Abe M, Yamaoka S, Sugiyama M (2011). Amino acids at positions 273 and 394 in rabies virus nucleoprotein are important for both evasion of host RIG-I-mediated antiviral response and pathogenicity. Virus Res.

[21] Nikolic J, Belot L, Raux H, Legrand P, Gaudin Y, Albertini AA (2018). Structural basis for the recognition of LDL-receptor family members by VSV glycoprotein. Nat Commun.

[22] Jamin M, Yabukarski F (2017). Nonsegmented Negative-Sense RNA Viruses-Structural Data Bring New Insights Into Nucleocapsid Assembly. Adv Virus Res.

[23] Masters PS, Banerjee AK (1988). Complex formation with vesicular stomatitis virus phosphoprotein NS prevents binding of nucleocapsid protein N to nonspecific RNA. J Virol.

[24] Mavrakis M, Méhouas S, Réal E, Iseni F, Blondel D, Tordo N, Ruigrok RW (2006). Rabies virus chaperone: Identification of the phosphoprotein peptide that keeps nucleoprotein soluble and free from non-specific RNA. Virology.

[25] Liu P, Yang J, Wu X, Fu ZF (2004). Interactions amongst rabies virus nucleoprotein, phosphoprotein and genomic RNA in virus-infected and transfected cells. J Gen Virol.

[26] Leyrat C, Schneider R, Ribeiro EA, Yabukarski F, Yao M, Gérard FC, Jensen MR, Ruigrok RW, Blackledge M, Jamin M (2012). Ensemble structure of the modular and flexible full-length vesicular stomatitis virus phosphoprotein. J Mol Biol.

[27] Gerard FCA, Ribeiro EdA, Leyrat C, Ivanov I, Blondel D, Longhi S, Ruigrok RWH, Jamin M (2009). Modular Organization of Rabies Virus Phosphoprotein. J Mol Biol.

[28] Yabukarski F, Leyrat C, Martinez N, Communie G, Ivanov I, Ribeiro EA, Buisson M, Gerard FC, Bourhis JM, Jensen MR (2016). Ensemble Structure of the Highly Flexible Complex Formed between Vesicular Stomatitis Virus Unassembled Nucleoprotein and its Phosphoprotein Chaperone. J Mol Biol.

[29] Gérard FCA, Bourhis J-M, Mas C, Branchard A, Vu DD, Varhoshkova S, Leyrat C, Jamin M (2022). Structure and Dynamics of the Unassembled Nucleoprotein of Rabies Virus in Complex with Its Phosphoprotein Chaperone Module. Viruses.

[30] Froger A, Hall JE (2007). Transformation of plasmid DNA into E. coli using the heat shock method. J Vis Exp.

[31] Studier FW (2005). Protein production by auto-induction in high density shaking cultures. Protein Expr Purif.

[32] Evans PR (2011). An introduction to data reduction: Space-group determination, scaling and intensity statistics. Acta Crystallogr D Biol Crystallogr.

[33] McCoy AJ, Grosse-Kunstleve RW, Adams PD, Winn MD, Storoni LC, Read RJ (2007). Phaser crystallographic software. J Appl Crystallogr.

[34] Emsley P, Lohkamp B, Scott WG, Cowtan K (2010). Features and development of Coot. Acta Crystallogr D Biol Crystallogr.

[35] Afonine PV, Grosse-Kunstleve RW, Echols N, Headd JJ, Moriarty NW, Mustyakimov M, Terwilliger TC, Urzhumtsev A, Zwart PH, Adams PD (2012). Towards automated crystallographic structure refinement with phenix.refine. Acta Crystallogr D Biol Crystallogr.

[36] Laskowski RA, Jabłońska J, Pravda L, Vařeková RS, Thornton JM (2018). PDBsum: Structural summaries of PDB entries. Protein Sci.

[37] Krissinel E (2012). Enhanced fold recognition using efficient short fragment clustering. J Mol Biochem.

[38] Nevers Q, Scrima N, Glon D, Le Bars R, Decombe A, Garnier N, Ouldali M, Lagaudrière-Gesbert C, Blondel D, Albertini A (2022). Properties of rabies virus phosphoprotein and nucleoprotein biocondensates formed in vitro and in cellulo. PLoS Pathog.

[39] Kirchdoerfer RN, Abelson DM, Li S, Wood MR, Saphire EO (2015). Assembly of the Ebola Virus Nucleoprotein from a Chaperoned VP35 Complex. Cell Rep.

[40] Guryanov SG, Liljeroos L, Kasaragod P, Kajander T, Butcher SJ (2016). Crystal Structure of the Measles Virus Nucleoprotein Core in Complex with an N-Terminal Region of Phosphoprotein. J Virol.

[41] Renner M, Bertinelli M, Leyrat C, Paesen GC, Saraiva de Oliveira LF, Huiskonen JT, Grimes JM (2016). Nucleocapsid assembly in pneumoviruses is regulated by conformational switching of the N protein. eLife.

[42] Jenni S, Horwitz JA, Bloyet L-M, Whelan SPJ, Harrison SC (2022). Visualizing molecular interactions that determine assembly of a bullet-shaped vesicular stomatitis virus particle. Nat Commun.

[43] Zhou K, Si Z, Ge P, Tsao J, Luo M, Zhou ZH (2022). Atomic model of vesicular stomatitis virus and mechanism of assembly. Nat Commun.

[44] Graham SC, Assenberg R, Delmas O, Verma A, Gholami A, Talbi C, Owens RJ, Stuart DI, Grimes JM, Bourhy H (2008). Rhabdovirus Matrix Protein Structures Reveal a Novel Mode of Self-Association. PLoS Pathog.

[45] Sievers F, Wilm A, Dineen D, Gibson TJ, Karplus K, Li W, Lopez R, McWilliam H, Remmert M, Söding J (2011). Fast, scalable generation of high-quality protein multiple sequence alignments using Clustal Omega. Mol Syst Biol.

[46] Ivanov I, Crépin T, Jamin M, Ruigrok RW (2010). Structure of the dimerization domain of the rabies virus phosphoprotein. J Virol.

[47] Donnelly CM, Cross EM, Forwood JK (2023). Crystal Structure of Rabies Virus (Strain CVS-11) P3 Dimerization Domain.

[48] Gupta AK, Blondel D, Choudhary S, Banerjee AK (2000). The phosphoprotein of rabies virus is phosphorylated by a unique cellular protein kinase and specific isomers of protein kinase. J Virol.

[49] Warrilow D, Fu ZF (2005). The World of Rhabdoviruses.

[50] Klein A, Eggerbauer E, Potratz M, Zaeck LM, Calvelage S, Finke S, Müller T, Freuling CM (2022). Comparative pathogenesis of different phylogroup I bat lyssaviruses in a standardized mouse model. PLoS Negl Trop Dis.

[51] Chenik M, Schnell M, Conzelmann KK, Blondel D (1998). Mapping the interacting domains between the rabies virus polymerase and phosphoprotein. J Virol.

[52] Jacob Y, Real E, Tordo N (2001). Functional interaction map of lyssavirus phosphoprotein: Identification of the minimal transcription domains. J Virol.

[53] Horwitz JA, Jenni S, Harrison SC, Whelan SPJ (2020). Structure of a rabies virus polymerase complex from electron cryomicroscopy. Proc Natl Acad Sci USA.

[54] Scrima N, Le Bars R, Nevers Q, Glon D, Chevreux G, Civas A, Blondel D, Lagaudrière-Gesbert C, Gaudin Y (2023). Rabies virus P protein binds to TBK1 and interferes with the formation of innate immunity-related liquid condensates. Cell Rep.

[55] Masatani T, Ozawa M, Yamada K, Ito N, Horie M, Matsuu A, Okuya K, Tsukiyama-Kohara K, Sugiyama M, Nishizono A (2016). Contribution of the interaction between the rabies virus P protein and I-kappa B kinase ϵ to the inhibition of type I IFN induction signalling. J Gen Virol.

[56] Fitzgerald KA, McWhirter SM, Faia KL, Rowe DC, Latz E, Golenbock DT, Coyle AJ, Liao S-M, Maniatis T (2003). IKKε and TBK1 are essential components of the IRF3 signaling pathway. Nat Immunol.

[57] Brzózka K, Finke S, Conzelmann K-K (2005). Identification of the Rabies Virus Alpha/Beta Interferon Antagonist: Phosphoprotein P Interferes with Phosphorylation of Interferon Regulatory Factor 3. J Virol.

[58] Lieu KG, Brice A, Wiltzer L, Hirst B, Jans DA, Blondel D, Moseley GW (2013). The Rabies Virus Interferon Antagonist P Protein Interacts with Activated STAT3 and Inhibits Gp130 Receptor Signaling. J Virol.

[59] Brzózka K, Finke S, Conzelmann K-K (2006). Inhibition of Interferon Signaling by Rabies Virus Phosphoprotein P: Activation-Dependent Binding of STAT1 and STAT2. J Virol.

[60] Vidy A, El Bougrini J, Chelbi-Alix MK, Blondel D (2007). The nucleocytoplasmic rabies virus P protein counteracts interferon signaling by inhibiting both nuclear accumulation and DNA binding of STAT1. J Virol.

[61] Deffrasnes C, Luo MX, Wiltzer-Bach L, David CT, Lieu KG, Wang LF, Jans DA, Marsh GA, Moseley GW (2021). Phenotypic Divergence of P Proteins of Australian Bat Lyssavirus Lineages Circulating in Microbats and Flying Foxes. Viruses.

[62] Blondel D, Regad T, Poisson N, Pavie B, Harper F, Pandolfi PP, De Thé H, Chelbi-Alix MK (2002). Rabies virus P and small P products interact directly with PML and reorganize PML nuclear bodies. Oncogene.

[63] Brice A, Whelan DR, Ito N, Shimizu K, Wiltzer-Bach L, Lo CY, Blondel D, Jans DA, Bell TDM, Moseley GW (2016). Quantitative Analysis of the Microtubule Interaction of Rabies Virus P3 Protein: Roles in Immune Evasion and Pathogenesis. Sci Rep.

[64] Moseley GW, Lahaye X, Roth DM, Oksayan S, Filmer RP, Rowe CL, Blondel D, Jans DA (2009). Dual modes of rabies P-protein association with microtubules: A novel strategy to suppress the antiviral response. J Cell Sci.

[65] Zhan J, Watts E, Brice AM, Metcalfe RD, Rozario AM, Sethi A, Yan F, Bell TDM, Griffin MDW, Moseley GW (2022). Molecular Basis of Functional Effects of Phosphorylation of the C-Terminal Domain of the Rabies Virus P Protein. J Virol.

