# Supplementary material for: Structural Determination of the Australian Bat Lyssavirus Nucleoprotein and Phosphoprotein Complex
Source: Viruses. Author manuscript; Available in PMC 2024 Jan 16. (PMC7615531; doi:10.3390/v16010033)
Supplement: Supplementary Material - Table S1 [file EMS193169-supplement-Supplementary_Material___Table_S1.pdf]

Table S1: List of atom-atom interactions across the nucleoprotein-phosphoprotein interface.

	Nucleoprotein				Phosphoprotein			
	Atom	Residue name	Residue number		Atom	Residue name	Residue number	Distance
Hydrogen bonds								
1	NH2	ARG	149	<-->	O	LEU	39	2.81
2	NH1	ARG	168		O	HIS	38	3.13
3	NH1	ARG	168		OE1	GLN	40	3.3
4	NH2	ARG	168		O	ALA	37	3.2
5	NH2	ARG	168		O	HIS	38	3.32
6	NH2	ARG	225		OD1	ASN	35	3.33
7	OH	TYR	233		ND2	ASN	31	2.95
8	O	THR	243		ND2	ASN	29	3
9	OG1	THR	243		OD1	ASN	29	3.09
10	N	ALA	253		OE1	GLU	22	3.14
11	N	ARG	254		OE2	GLU	22	2.94
12	O	PHE	261		N	Val	6	2.8
13	N	LYS	263		O	ILE	4	3.08
14	NH2	ARG	270		O	GLY	14	3.14
15	OG1	THR	279		OG1	THR	24	2.78
16	OE2	GLU	403		NH1	ARG	12	2.71
17	OE2	GLU	403		NH2	ARG	12	2.69
Salt bridges								
1	NZ	LYS	247		OE2	GLU	33	2.51
2	NH2	ARG	270		OD2	ASP	17	3.8
3	OE2	GLU	403		NH2	ARG	12	2.69
